# Retrospective Analysis of the Clinical and Radiological Outcomes Following Modified Dunn Osteotomy in Patients with Stable, Moderate-to-severe Chronic Slipped Capital Femoral Epiphysis

**DOI:** 10.1055/s-0044-1788672

**Published:** 2024-12-21

**Authors:** Basant Kumar Bhuyan

**Affiliations:** 1 Departamento de Ortopedia e Traumatologia, Faculty of Medicine, GMERS Medical College and General Hospital, Himmatnagar, Sabarkantha, Gujarat, Índia

**Keywords:** hip, osteonecrosis, osteotomy, slipped capital femoral epiphysis

## Abstract

**Objective** The management of slipped capital femoral epiphysis (SCFE) has been completely transformed by modified Dunn osteotomy, a subcapital realignment osteotomy achieved through a safe surgical dislocation technique originally described by Ganz. The purpose of this study was to evaluate the clinical and radiological outcomes of patients with moderate to severe SCFE after modified Dunn osteotomy.

**Methods** A total of 15 patients (16 hips, with one bilateral case; 12 males, 3 females) aged from 10.2 to 17-years-old (mean: 14.3) with stable, moderate to severe, chronic SCFE (moderate: = 6; severe = 10) underwent modified Dunn osteotomy as treatment. The hip joint range of motion (ROM), Harris (HHS), and Merle d'Aubigné (MdA) hip scores were used for clinical assessments. They were assessed radiographically using the Southwick and Alpha angles.

**Results** At the most recent follow-up (mean 8.6 years; 3.1–14), the mean hip joint ROM, the mean HHS (preoperative: 69.20 ± 5.94; postoperative: 86 ± 7.37; *p* < 0.00001), and the mean MdA score (preoperative: 12.47 ± 1.13; postoperative: 14.27 ± 1.83; *p* < 0.00001) all showed statistically significant clinical improvements The radiological results demonstrated improvements in the mean Southwick angle (preoperative: 56.60 ± 12.89°; postoperative: 16.40 ± 4.69°; *p* < 0.00001) and Alpha angle (preoperative: 101.87 ± 12.88°; postoperative: 29.33 ± 7.29°; *p* < 0.00001). There were two significant postoperative complications identified: femoral head avascular necrosis (AVN) and deep infection.

**Conclusion** According to the study's findings, the modified Dunn osteotomy is a safe, efficient treatment option for stable moderate-to-severe chronic SCFE with a manageable risk of complications.

## Introduction

Slipped capital femoral epiphysis (SCFE) is a well-known debilitating hip condition in children and young adults with a global incidence ranging from 0.33 to 24.58 per 100,000 people between the ages of 8 and 15 years, depending on gender and ethnicity. 1 Proximal femoral deformity in SCFE results from the migration and displacement of the capital femoral epiphysis from the metaphysis through the physeal plate in a postero-inferior direction.

According to the length of symptoms, the stability of the slip and the magnitude of the slip three distinct classification systems are universally accepted. The severity of SCFE can be classified as acute (lasting less than 3 weeks), chronic (more than 3 weeks), or acute and chronic (more than 3 weeks with exacerbation of symptoms). 2 Based on its capacity to support weight with or without assistance, the classification categorizes SCFE into stable and unstable types. 3 By using the Southwick method which splits the slip angle into mild (< 30°), moderate (30–60°), and severe (> 60°) degrees to measure the radiological magnitude of the slip. 4

The primary objectives of treating SCFE are preventing additional slip progression, stabilizing and reinstating hip function, preventing early hip osteoarthritis (OA), and reducing the risk of avascular necrosis (AVN). There has been discussion about the best course of treatment for SCFE, but disagreements still persist between conventional in situ pinning and more aggressive reconstructive procedures. Femoroacetabular impingement (FAI) is the result of an aberrant head–neck offset in moderate to severe cases of SCFE after in situ pinning, where the hip remains malformed despite some remodelling. 5 Furthermore, it is the primary cause of mechanical derangement of the hip joint, which results in cartilage damage and labral damage, predisposing to the early development of secondary hip OA. 6

A number of distinct realignment osteotomy techniques have been reported at the subcapital, basicervical, intertrochanteric, and subtrochanteric levels to enhance the long-term prognosis of patients with SCFE. 7 8 9 10 11 The Dunn procedure, a realignment osteotomy carried out at the sub-capital level, offers the greatest amount of correction. However, it carries a possible risk of AVN that may be caused by iatrogenic damage to the blood supply of the proximal femoral epiphysis. For the treatment of SCFE, Leunig et al. 12 invented subcapital osteotomy, by using a safe surgical hip dislocation approach developed by Ganz et al. 13 The capital femoral head vascularity is preserved by this modified Dunn osteotomy, which enables the identification and preservation of the retinacular vessels through meticulous preparation of an extended retinacular flap. 13 The purpose of this study was to assess the clinical and radiological results and complications of patients treated for stable, moderate to severe, chronic SCFE with this procedure, and to compare these findings with those of similar published series.

## Materials and Methods



**Video 1** Evaluation of epiphyseal perfusion after capital realignment osteotomy.


The present work was approved by the institutional committee under the Independent Ethics Committee (IEC) number /49/01/09/2023.

All teenage patients with stable, moderate-to-severe, chronic SCFE and open physis who presented between January 2010 and December 2020 were included in this retrospective case series. The exclusion criteria were acute, acute on chronic, or unstable SCFE, as well as other related congenital or acquired hip deformities, and any history of previous hip surgery. The index procedure was carried out with the full informed written consent of all parents.

**Fig. 1:**
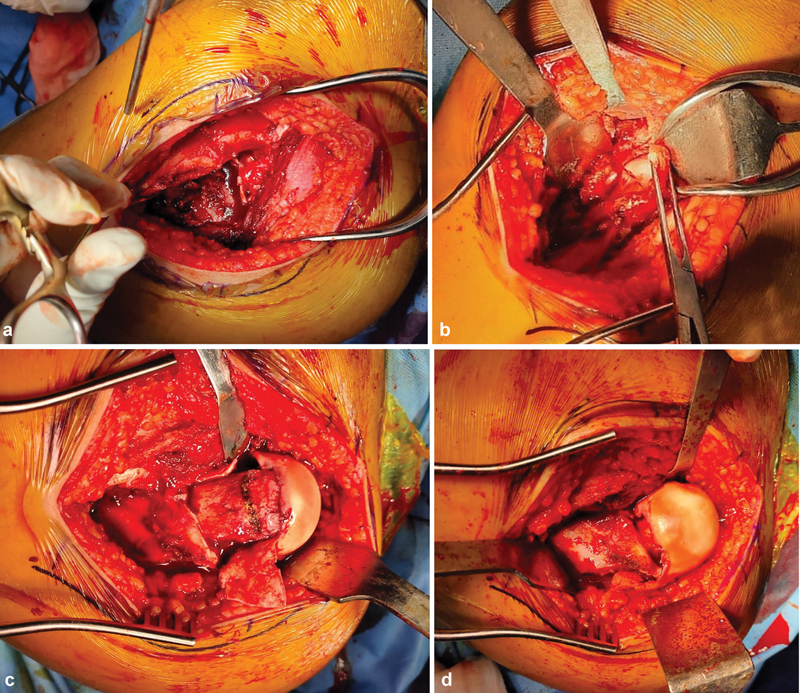
Hip joint is exposed through ( **a** ) trochanteric osteotomy ( **b** ) capsulotomy. ( **c** ) Subcapital osteotomy area is marked. ( **d** ) Subcapital realignment without tension.

The modified Dunn osteotomy procedure was performed using the operative technique outlined by Leunig et al. 12 Following a trochanteric flip osteotomy and Z-shaped capsulotomy, the hip joint was exposed through the Gibson interval ( Fig. 1a and b ). After the ligamentum teres was excised and the femoral epiphysis was temporarily fixed with K-wires, safe surgical dislocation of the hip was carried out. The stable portion of the greater trochanter was carefully trimmed on its posterior and superior aspects to the size of the femoral neck, and an extended retinacular soft tissue flap was created, housing the retinacular vessels up to the capital femoral epiphysis. A cautious and controlled mobilization of the epiphysis was attempted following resection of the posteromedial metaphyseal callus. After careful femoral neck trimming, which did not strain the vessels, a capital realignment was performed ( Fig. 1c and d ). Femoral head perfusion was evaluated both during surgical dislocation and following a capital realignment procedure ( Video 1 ). There were two 6.5-mm partially threaded cannulated screws used to precisely fix the epiphysis, and two 4 mm cannulated cancellous screws used to reattach the osteotomized greater trochanter.

Information was gathered from the hospital medical records database about demographics, clinical characteristics, surgical technique, intraoperative findings, type of fixation, postoperative follow-up, and complications. The range of motion (ROM) of the hip joint, encompassing internal and external rotation with 90° of hip flexion, was measured for both preoperative and postoperative functional assessments. Using questionnaires based on the Harris (HHS) and Merle d'Aubigné (MdA) hip scores, subjective outcomes were evaluated. 14 15 Anteroposterior pelvis and Lauenstein frog-leg lateral view radiographs are used in radiological evaluation to measure the Southwick slip angle and Alpha angle. 4 16 These values were computed and compared to the preoperative values.

### Statistical Analysis

The means and standard deviations were used to represent the values of the radiographic measurements and the clinical assessment scores on the various scales. The pre- and postoperative values were compared using paired *t* tests. A *p* -value lower than 0.05 was considered statistically significant. The Statistical Package for Social Science (SPSS, IBM Corp., New York, USA), version 27.0 for Microsoft Windows, was used for statistical analysis.

## Results

The data of 15 patients (16 hips) with an average age of 14.3 ± 1.8 (range, 10.2–17) years at the time of surgery were retrospectively reviewed. Of these, 12 (80%) were male and 3 (20%) were female. The age distribution was of 12.5 ± 1.6 (7–17) years for the male patients and 10.8 ± 1.3 (9–13) for the females. Every slip met the stable variety classification criteria established by Loder and the chronicity criteria established by Fahey and O'Brien. According to the Southwick classification of slip angle, 10 hips (62.5%) had a severe degree of slip, with a mean slip angle of 66.1 ± 13.9° (61–76°), and 6 hips (37.5%) had a moderate degree of slip, with a mean slip angle of 42.3 ± 6.3° (39–45°). There were 9 affected hips (56.2%) on the right side and 7 (43.7%) on the left side, with one patient with bilateral involvement.

Table 1 displays the patients' demographic and clinical information, such as their stability, degree of Southwick's slip angle, and chronicity of symptoms.

**Table 1:** Patient characteristics

Case nr.	Gender	Age (years)	Side	Duration (weeks)	Fahey and O'Brien Classification	Loder classification	Southwick classification	Follow-up (years)
1	Male	10.2	Left	4	Chronic	Stable	Moderate	14
2	Male	15.3	Both	12	Chronic	Stable	Severe	13.2
3	Male	14.5	Right	9	Chronic	Stable	Severe	12.4
4	Female	11.8	Left	8	Chronic	Stable	Severe	11.7
5	Male	14.6	Right	6	Chronic	Stable	Moderate	11
6	Male	15.7	Right	10	Chronic	Stable	Severe	10.5
7	Male	17	Left	8	Chronic	Stable	Severe	9.7
8	Female	12.8	Right	9	Chronic	Stable	Severe	9
9	Male	13.6	Right	4	Chronic	Stable	Moderate	8.1
10	Male	14.4	Left	5	Chronic	Stable	Moderate	7.2
11	Male	16	Right	6	Chronic	Stable	Severe	6
12	Female	13.6	Left	4	Chronic	Stable	Moderate	5.6
13	Male	15	Right	11	Chronic	Stable	Severe	4.2
14	Male	14.7	Right	5	Chronic	Stable	Moderate	3.9
15	Male	16.2	Left	12	Chronic	Stable	Severe	3.1

A mean follow-up period of 8.6 (3.1–14) years was observed for all patients. The hip ROM at the final follow-up significantly improved with respect to mean hip flexion, internal (IR) and external (ER) rotation in 90° of flexion. After surgery, the average flexion angle was 91 ± 17.24° (65–120°), the average IR angle was 28 ± 6.5° (15–30°), and the average ER angle was 27.3 ± 5.3° (20–35°).

According to the HHS evaluation results, 4 patients had excellent clinical outcomes (≥ 90 points), 7 good (80–90), and one each had a fair (70–80) and poor (< 70) outcome. Preoperatively, the average HHS was 69.2 ± 5.9 (61–78); postoperatively, it improved to 86 ± 7.3 (75–95), with *p* < 0.00001.

The postoperative MdA score were rated as good (15–17) in 8 patients, fair (13 or 14) in 6, and poor (<13) in 2 patients. At the most recent follow-up, the mean MdA score improved from 12.47 ± 1.13 (11–14) points preoperatively to 14.27 ± 1.83 (10–17) postoperatively, with *p* < 0.00001.

**Fig. 2:**
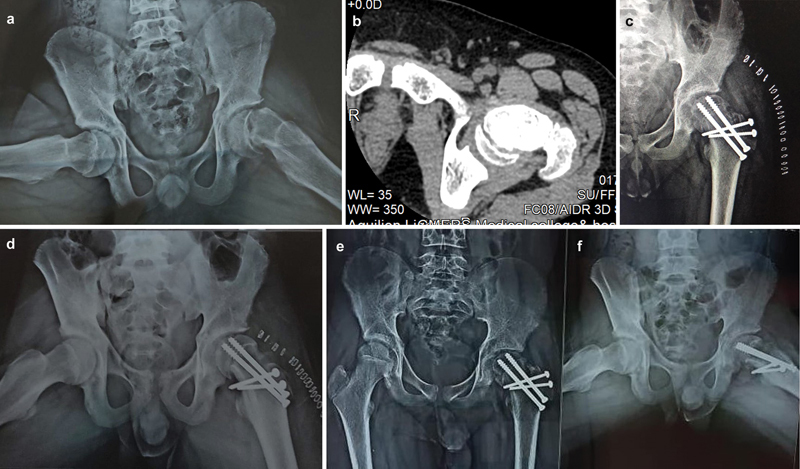
( **a** ) Preoperative radiograph of the 14.4-year-old boy's frog leg; lateral view demonstrating moderate degree slip on the left hip. ( **b** ) Axial CT image showing displacement of capital femoral epiphysis. ( **c–d** ) Anteroposterior and frog leg lateral view immediately after surgery. ( **e–f** ) Anteroposterior and frog leg lateral view radiographs taken 4.3 years after a modified Dunn osteotomy.

The radiographic anatomy of the proximal femur improved in every patient after the modified Dunn osteotomy procedure. Between the pre- and postoperative periods, the mean Southwick slip angle improved from 56.60 ± 12.86° (39–76°) to 16.40 ± 4.69° (5–25°), with *p* < 0.00001. Similarly, the mean Alpha angle improved from 101.87 ± 12.88° (85–125°) to 29.33 ± 7.29° (20–40°), with *p* < 0.00001. Patient No.10 ( Fig. 2a–f ) had good to excellent radiographic and clinical outcomes.

Table 2 summarizes the clinical and radiological parameter evaluations performed both prior to surgery and at the most recent follow-up visit.

**Table 2:** Clinical and radiographic outcomes

Parameter	Preoperative (mean)	Postoperative (mean)	Mean correction	*p* -value	*t* -value	Complications
Clinical results						AVN ( *n* = 1), deep infection ( *n* = 1)
ROM						
Flexion	61.67	91.00	29.33	< 0.00001	15.59	
ER in flexion	52.67	27.33	25.34	< 0.00001	12.42	
IR in flexion	0	28.00	28	< 0.00001	16.7	
HHS	69.20	86	16.8	< 0.00001	19.85	
MdA score	12.47	14.27	1.8	< 0.00001	6.87	
Radiographic results						
Southwick slip angle	56.60	16.40	40.2	< 0.00001	-15.07	
Alpha angle	101.87	29.33	72.54	< 0.00001	-36.1	

**Abbreviations:** ER, external rotation; HHS, Harris hip score; IR, internal rotation; MdA, Merle d' Aubignè score; ROM, range of motion.

**Fig. 3:**
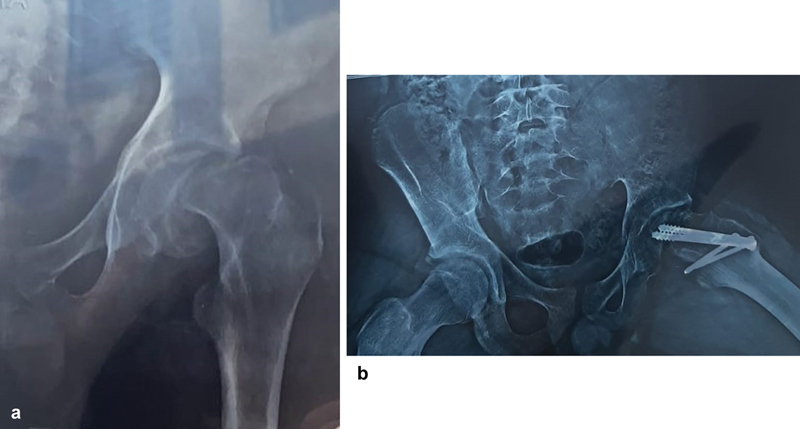
( **a** ) Anteroposterior view radiograph of a 15.3-year-old boy with severe SCFE on the left side taken prior to surgery. ( **b** ) Following a modified Dunn osteotomy, a 2.5-year follow-up radiograph revealed signs of AVN left hip joint.

Deep infection and AVN of the femoral head were the two main postoperative complications. A deep infection that was eventually treated with debridement and exploration resulted in a stiff hip joint. A patient who had bilateral severe SCFE and hypothyroidism experienced a poor outcome after developing AVN of the left hip joint and partial femoral head collapse 12 weeks after surgery ( Fig. 3a and b ). He was counselled to schedule routine check-ups and THR following skeletal maturity. No other patients had any records of chondrolysis, heterotopic ossification (HO), implant failure, FAI symptoms, or early OA following surgery.

## Discussion

The natural history of SCFE has shown that as the degree of slip increases, there is a noticeable and progressive deterioration of the hip joint. 17 The FAI results from altered proximal femoral anatomy caused by anterolateral metaphyseal prominence. This has been linked to chondrolabral damage, reduced ROM, increased hip pain, and early hip OA. 18

Significant advancements in surgical techniques have occurred over time, and more aggressive joint reconstructive procedures have recently emerged to preserve femoral head vascularity in SCFE while offering greater anatomic correction of the deformity. Restoring normal hip function while addressing residual metaphyseal deformity and physeal stability has been reported to be a potential positive outcome of the modified Dunn osteotomy.

This procedure was first described by Leunig et al. using a safe surgical dislocation technique that allowed for ongoing intraoperative monitoring of the epiphyseal blood supply. All 30 patients had successful anatomical reduction and no AVN of the femoral head, according to their positive reports. 19

Ziebarth et al. 20 followed 40 patients from two institutions with moderate to severe SCFE for 1 or 3 years. With a modified Dunn procedure, they were able to replicate the 0% rate of AVN or chondrolysis and reported a good success rate.

Using a modified Dunn procedure, Madan et al. 21 reported the treatment of 28 patients with severe SCFE. The mean modified HHS at the final follow-up (mean: 38.6 months), was 89.1, and the mean nonarthritic hip score (NAHS) was 91.3. At the final follow-up, the lateral slip angle was corrected by a mean of 50.9°, and the ROM was almost normal. Furthermore, two patients had AVN that was proven to have existed beforehand, and another two acquired it after surgery. Nonunion, implant failure, infection, DVT, or HO were not observed in any of the patients.

Among the 43 SCFE patients treated by Upasani et al., 22 37 (86%) experienced severe slip; 16 (37%) experienced 22 complications; 10 (23%) experienced AVN of the femoral head; 4 (9%) experienced femoral neck nonunion necessitating revision surgery; and 2 (5%) experienced hip dislocation following surgery.

Abdelazeem et al. 23 conducted a prospective study after the modified Dunn procedure on 31 patients (32 hips) with moderate to severe SCFE. Following treatment, all clinical and radiological measurements showed a significant improvement at a mean follow-up of 24.1 months. Major complications following surgery included one case of postoperative AVN with complete femoral head collapse and arthritis, as well as one case of deep infection.

A prospective case series with 30 patients (32 hips) with stable chronic SCFE was reported by Elmarghany et al. 24 A total of 30 hips (94%) had good and excellent clinical and radiographic outcomes over a mean follow-up period of 14.5 months with regard to radiographic parameters and hip function. However, 3 patients experienced postoperative AVN, 1 experienced a poor reduction that required revision, and 1 experienced a postoperative deep infection. Their clinical outcome results ranged from fair to poor.

A modified Dunn procedure was used by Lerch et al. 25 to treat 46 patients (46 hips) with severe SCFE. Of that total, 2 hips (5%) experienced AVN of the femoral head, requiring additional surgery; 3 (7%) experienced implant revision; and 1 (2%) experienced OA progression. At the 10-year follow-up, the cumulative survival rate was 86%.

Ebert et al. 26 used a modified Dunn procedure to treat 15 patients who had severe slip SCFE. Within that total, 8 patients achieved good results at a mean follow-up of 3.8 years, according to both clinical and functional outcome analysis (HHS >80). In 10 patients, the Nottingham health profile (NHP) measured quality of life, which was deemed good. There was AVN occurrence in 4 (26%) of the 15 patients.

Zuo et al. 27 evaluated the results of 20 patients (21 hips) with severe SCFE in retrospect. From that total, 19 patients showed excellent clinical and radiographic outcomes in terms of hip function and radiographic parameters at a mean follow-up of 31.2 months. However, 1 patient (5%) developed an implant failure experienced a poor outcome. There were no cases of AVN, FAI, OA, growth plate closure, HO, trochanteric nonunion, or disparity in limb length noted.

Using a modified Dunn technique, Agashe et al. 28 treated 30 consecutive hips with moderate and severe SCFE. The HHS mean follow-up functional outcome at 25.36 months indicated that all the operated hips had nearly normal ROMs, with results being excellent in 6 patients, good in 17, fair in 6, and poor in 1 patient. Furthermore, one hip experienced postoperative subluxation, which was rectified through revision surgery, while 2 hips (6.6%) developed AVN.

Table 3 lists the studies' references, along with the number of cases, classifications, duration of follow-up, postoperative clinical and radiological outcomes, incidence of AVN, and other complications.

**Table 3:** Comparison of the included studies data and outcomes

Author (year)	Number of hips	Type of SCFE (moderate/severe)	Length of follow-up (years)	Postoperative clinical results	Postoperative radiological results	AVN of femoral head	Complications
Ziebarth et al. 20 (2009)	40	16/19 (5 Hips - no information)	2.6	HHS: 99.6 WOMAC: 1.2 (pain), 3 (function) MdA: 17.8	Slip angle:8.6 ° Alpha angle: 40.6°	0%	HO: 2.5% Residual impingement: 2.5% Delayed union: 7.5% Implant failure: 7.5%
Madan et al. 21 (2013)	28	0/28	3.2	Modified HHS: 89.1 NAHS: 91.3	Slip angle: 8°	7.1%	Condrolysis: 3.6%
Upasani et al. 22 (2014)	43	6/37	2.6	Dindo-Clavien classification Grading: I - 4.6% II - 6.9% III - 34.5% IV - 4.6%	−	23%	Femoral neck nonunion: 9% Hip dislocation: 5%
Abdelazeem et al. 23 (2016)	32	10/22	2Y	HHS: 96.3 WOMAC: 97 MdA: 16.7	Slip angle: 12.2° Alpha angle: 46.9°	3.1%	Deep infection: 3.1%
Elmarghany et al. 24 (2017)	32	11/21	1.2	HHS: 96.2 WOMAC: 3.3 MdA: 16.8 Heyman and Herdon outcome: excellent or good - 93.7%	Slip angle: 5.6° Alpha angle: 51.1°	9.3%	Deep infection: 3% Revision: 3%
Lerch et al. 25 (2019)	46	0/46	9	HHS: 94 MdA: 17 HOOS: 91	Slip angle: 7°	5%	OA: 2%, Implant failure: 7%
Ebert et al. 26 (2019)	15	0/15	3.8	HHS: 85.7 NHP: 0.91 VAS: 1.6	−	26.6%	Hip subluxation: 13.3% Implant failure: 13.3%
Zuo et al. 27 (2020)	21	0/21	2.7	HHS: 96.7 WOMAC: 95.4	Slip angle: 7.5° alpha angle: 42°	0%	Implant failure: 5%
Agashe et al. 28 (2020)	30	20/10	2.1	HHS: 81.8	Slip angle: 9.9° Alpha angle: 33.6°	6.6%	Hip subluxation: 3.3%
Present series	16	6/10	8.6	HHS: 86 MdA: 14.2	Slip angle: 16.4° Alpha angle: 29.3°	6.2%	Deep infection: 6.2%

**Abbreviations:** AVN, avascular necrosis; HHS, Harris hip score; HO, heterotopic ossification; HOOS, hip disability and osteoarthritis outcome score; MdA, Merle d' Aubignè score; NAHS, nonarthritic hip score; NHP, Nottingham health profile; VAS, visual analogue scale; WOMAC, Western Ontario and McMaster Universities arthritis index.

The number of patients in this study who were treated with the modified Dunn osteotomy procedure for moderate to severe SCFE was similar to the number reported in the literature. At the most recent follow-up, two complications were found (AVN, deep infection), along with notable improvements in ROM, mean HHS, and mean MdA. According to these findings, a modified Dunn osteotomy technique can be used to realign SCFE with an open physis and a low risk of complications.

For the treatment of moderate to severe forms of SCFE, modified Dunn osteotomy is a safe and reliable surgical technique that should be strongly considered. With a manageable complication rate, this procedure can restore the proximal femoral anatomy by addressing the slip angle and head-neck offset, helping to maintain normal hip function. 29

This study's strengths include the use of a well-defined patient population with moderate to severe SCFE and postoperative functional and radiological assessment results that are positively correlated with successful long-term results. This study has several limitations, such as its small sample size, retrospective single cohort study design, potential selection bias due to lack of randomization, and noncomparative design without a control group.

## Conclusion

The modified Dunn osteotomy, by meticulously preserving the posterior retinacular soft tissue flap, aids in the anatomical realignment of a displaced capital femoral epiphysis while maintaining femoral head perfusion. The successful execution of this intricate reconstructive procedure with favorable long-term results requires a precise understanding of the hip's vascular anatomy and a thorough understanding of the surgical technique.
