# Supplementary material for: Retrospective Analysis of the Clinical and Radiological Outcomes Following Modified Dunn Osteotomy in Patients with Stable, Moderate-to-severe Chronic Slipped Capital Femoral Epiphysis
Source: Rev Bras Ortop (Sao Paulo). 2024 Dec 21;59(6):e913–21. doi: 10.1055/s-0044-1788672 (PMC11663068; doi:10.1055/s-0044-1788672)
Supplement: Related texts

# Research article: Análise retrospectiva dos desfechos clínicos e radiológicos após a osteotomia de Dunn modificada em pacientes com escorregamento epifisário proximal do fêmur estável, crônico e moderado a grave

**Keywords:** escorregamento das epífises proximais do fêmur, osteonecrose, osteotomia, quadril

## Abstract

**Objetivo** O manejo do escorregamento epifisário proximal do fêmur (EEPF) foi completamente transformado pela osteotomia de Dunn modificada, uma osteotomia de realinhamento subcapital por meio da técnica segura de luxação cirúrgica originalmente descrita por Ganz. O objetivo deste estudo foi avaliar os desfechos clínicos e radiológicos de pacientes com EEPF moderado a grave após a osteotomia de Dunn modificada.

**Métodos** Um total de 15 pacientes (16 quadris, com um caso bilateral; 12 homens, 3 mulheres) com idade entre 10,2 e 17 anos (média: 14,3) e EEPF crônico, estável e moderado a grave (moderado = 6; grave = 10) foram submetidos à osteotomia de Dunn modificada como tratamento. A amplitude de movimento (ADM) da articulação do quadril e os escores de quadril de Harris (HHS) e Merle d'Aubigné (MdA) foram utilizados para avaliações clínicas. A avaliação radiográfica usou os ângulos de Southwick e alfa.

**Resultados** No acompanhamento mais recente (média: 8,6 anos; 3,1–14), a média da ADM da articulação do quadril, o HHS médio (pré-operatório: 69,20 ± 5,94; pós-operatório: 86 ± 7,37, *p* < 0,00001) e o MdA médio (pré-operatório: 12,47 ± 1,13; pós-operatório: 14,27 ± 1,83, *p* < 0,00001) demonstraram melhoras clínicas estatisticamente significativas. Os resultados radiológicos demonstraram melhoras no ângulo Southwick médio (pré-operatório: 56,60 ± 12,89°; pós-operatório: 16,4 ± 4,69°, *p* < 0,00001) e no ângulo alfa (pré-operatório: 101,87 ± 12,88°; pós-operatório: 29,33 ± 7,29°, *p* < 0,00001). Foram observadas duas complicações pós-operatórias significativas: necrose avascular (NAV) da cabeça femoral e infecção profunda.

**Conclusão** De acordo com os achados do estudo, a osteotomia de Dunn modificada é uma opção terapêutica segura e eficaz no EEPF estável, crônico e moderado a grave, com risco controlável de complicações.

## Introdução

O escorregamento epifisário proximal do fêmur (EEPF) é uma doença debilitante e bem conhecida do quadril em crianças e adultos jovens, com incidência global de 0,33 a 24,58 por 100.000 pessoas entre 8 e 15 anos de idade, dependendo do sexo e da etnia. 1 A deformidade femoral proximal no EEPF é causada pela migração e deslocamento da epífise femoral capital da metáfise através da placa fisária em direção póstero-inferior.

De acordo com a duração dos sintomas e a estabilidade e magnitude do escorregamento, três sistemas de classificação distintos são universalmente aceitos. A gravidade do EEPF pode ser classificada como aguda (duração inferior a 3 semanas), crônica (superior a 3 semanas) ou crônica e aguda (duração superior a 3 semanas com exacerbação dos sintomas). 2 Com base na capacidade de suportar peso com ou sem assistência, a classificação categoriza o EEPF em tipos estáveis e instáveis. 3 O método de Southwick divide o ângulo de deslizamento em brando (< 30°), moderado (30–60°) e grave (> 60°) para medir a magnitude radiológica do escorregamento. 4

Os objetivos primários do tratamento do EEPF são prevenir a maior progressão do escorregamento, estabilizar o quadril e restabelecer sua função, prevenir a osteoartrite (OA) precoce desta articulação e reduzir o risco de necrose avascular (NAV). Discute-se o melhor tratamento para o EEPF, mas ainda há divergências entre a fixação in situ convencional e os procedimentos mais agressivos de reconstrução. O impacto femoroacetabular (IFA) é decorrente do deslocamento aberrante da cabeça e do colo em casos moderados a graves de EEPF após a fixação in situ, em que o quadril continua a apresentar malformação apesar de algum remodelamento. 5 Além disso, essa é a principal causa de distúrbio mecânico da articulação do quadril, o que leva a danos cartilaginosos e labrais, predispondo ao desenvolvimento precoce de OA secundária do quadril. 6

Diversas técnicas distintas de osteotomia de realinhamento foram relatadas nos níveis subcapital, basicervical, intertrocantérico e subtrocantérico para melhorar o prognóstico em longo prazo de pacientes com EEPF. 7 8 9 10 11 O procedimento de Dunn, uma osteotomia de realinhamento realizada no nível subcapital, oferece a maior correção. Entretanto, apresenta um possível risco de NAV, que pode ser causada por dano iatrogênico ao suprimento sanguíneo da epífise femoral proximal. Para o tratamento da EEPF, Leunig et al. 12 inventaram a osteotomia subcapital, utilizando a abordagem segura de luxação cirúrgica do quadril desenvolvida por Ganz et al. 13 A vascularização da cabeça femoral é preservada por esta osteotomia de Dunn modificada, que permite a identificação e preservação dos vasos retinaculares por meio da preparação meticulosa de um retalho retinacular estendido. 13 O objetivo deste estudo foi avaliar os desfechos clínicos e radiológicos e as complicações de pacientes com EEPF estável crônico moderado a grave submetidos a este procedimento e comparar esses achados com aqueles de séries semelhantes publicadas.

## Materiais e Métodos

O presente estudo foi aprovado pelo comitê institucional sob o Comitê Independente de Ética (IEC), número /49/01/09/2023.

Todos os pacientes adolescentes com EEPF estável crônico moderado a grave e fises abertas atendidos entre janeiro de 2010 e dezembro de 2020 foram incluídos nesta série retrospectiva de casos. Os critérios de exclusão foram EEPF agudo, crônico agudizado, ou instável, assim como outras deformidades congênitas ou adquiridas do quadril relacionadas e qualquer histórico de cirurgia anterior no quadril. O procedimento índice foi realizado após a assinatura do termo de consentimento livre e esclarecido pelos pais dos pacientes.

O procedimento de osteotomia modificada de Dunn foi realizado utilizando a técnica operatória descrita por Leunig et al. 12 Após uma osteotomia trocantérica e capsulotomia em forma de Z, a articulação do quadril foi exposta pelo intervalo de Gibson ( Fig. 1a, b ). Depois da excisão do ligamento redondo e a fixação temporária da epífise femoral com fios de Kirschner, a luxação cirúrgica segura do quadril foi realizada. A porção estável do trocanter maior foi cuidadosamente aparada em suas faces posterior e superior ao tamanho do colo femoral, e um retalho retinacular estendido de tecido mole, abrigando os vasos retinaculares, foi criado até a epífise da cabeça femoral. Uma mobilização cautelosa e controlada da epífise foi tentada após a ressecção do calo metafisário posteromedial. Após cuidadoso corte do colo femoral, sem atingir os vasos, foi realizado o realinhamento capital ( Fig.1 c, d ). A perfusão da cabeça femoral foi avaliada durante a luxação cirúrgica e depois do procedimento de realinhamento capital ( Vídeo 1 ). Dois parafusos canulados com rosca parcial de 6,5 mm foram utilizados para fixação precisa da epífise e dois parafusos esponjosos canulados de 4 mm foram usados para recolocar o trocânter maior osteotomizado.

**Vídeo 1** Avaliação da perfusão epifisária após osteotomia de realinhamento capital.


**Fig. 1:** . A articulação do quadril é exposta por meio de ( **a** ) osteotomia trocantérica e ( **b** ) capsulotomia. ( **c** ) Marcação da área de osteotomia subcapital. ( **d** ) Realinhamento subcapital sem tensão.

As informações foram coletadas do banco de dados de prontuários hospitalares e incluíram dados demográficos, características clínicas, técnica cirúrgica, achados intraoperatórios, tipo de fixação, acompanhamento pós-operatório e complicações. A amplitude de movimento (ADM) da articulação do quadril, abrangendo rotação interna e externa com 90° de flexão da articulação, foi medida para avaliações funcionais pré e pós-operatórias. Os resultados objetivos dos escores de quadril de Harris (HHS) e Merle d'Aubigné (MdA) foram avaliados. 14 15 Radiografias anteroposteriores da pelve e em perna de rã de Lauenstein foram usadas na avaliação radiológica para medida do ângulo de deslizamento de Southwick e do ângulo alfa. 4 16 Esses valores foram computados e comparados aos valores pré-operatórios.

## Análise Estatística

Médias e desvios-padrão representaram os valores das medidas radiográficas e os escores da avaliação clínica nas diversas escalas. Os valores pré- e pós-operatórios foram comparados por meio de testes *t* pareados. Um valor de *p* inferior a 0,05 indicou significância estatística. O software Statistical Package for Social Science (SPSS, IBM Corp., Nova Iorque, EUA), versão 27.0 para Microsoft Windows, foi utilizado para análise estatística.

## Resultados

Os dados de 15 pacientes (16 quadris) com idade média de 14,3 ± 1,8 (10,2–17) anos no momento da cirurgia foram revisados de forma retrospectiva. Destes pacientes, 12 (80%) eram do sexo masculino e três (20%) do feminino. A distribuição etária foi de 12,5 ± 1,6 anos para os pacientes do sexo masculino (7–17) e 10,8 ± 1,3 anos para as do feminino (9–13). Cada escorregamento atendeu aos critérios de classificação de variedade estável estabelecidos por Loder e aos critérios de cronicidade estabelecidos por Fahey e O'Brien. De acordo com a classificação de ângulo de deslizamento de Southwick, 10 quadris (62,5%) apresentaram grau grave de escorregamento, com ângulo de escorregamento médio de 66,1 ± 13,9° (61–76°) e 6 quadris (37,5%) apresentaram grau de escorregamento moderado, com ângulo de escorregamento médio de 42,3 ± 6,3° (39–45°). O acometimento ocorreu do lado direito em 9 quadris (56,2%) do lado esquerdo em 7 (43,7%), sendo bilateral em um paciente.

A Tabela 1 mostra informações demográficas e clínicas dos pacientes, como estabilidade, grau de ângulo de deslizamento de Southwick e cronicidade dos sintomas.

**Tabela 1:** Características dos pacientes

Número do caso	Sexo	Idade (anos)	Lado	Duração (semanas)	Classificação de Fahey e O'Brien	Classificação de Loder	Classificação de Southwick	Acompanhamento (anos)
1	Masculino	10,2	Esquerdo	4	Crônica	Estável	Moderada	14
2	Masculino	15,3	Ambos	12	Crônica	Estável	Grave	13,2
3	Masculino	14,5	Direito	9	Crônica	Estável	Grave	12,4
4	Feminino	11,8	Esquerdo	8	Crônica	Estável	Grave	11,7
5	Masculino	14,6	Direito	6	Crônica	Estável	Moderada	11
6	Masculino	15,7	Direito	10	Crônica	Estável	Grave	10,5
7	Masculino	17	Esquerdo	8	Crônica	Estável	Grave	9,7
8	Feminino	12,8	Direito	9	Crônica	Estável	Grave	9
9	Masculino	13,6	Direito	4	Crônica	Estável	Moderada	8,1
10	Masculino	14,4	Esquerdo	5	Crônica	Estável	Moderada	7,2
11	Masculino	16	Direito	6	Crônica	Estável	Grave	6
12	Feminino	13,6	Esquerdo	4	Crônica	Estável	Moderada	5,6
13	Masculino	15	Direito	11	Crônica	Estável	Grave	4,2
14	Masculino	14,7	Direito	5	Crônica	Estável	Moderada	3,9
15	Masculino	16,2	Esquerdo	12	Crônica	Estável	Grave	3,1

O período médio de acompanhamento foi de 8,6 (3,1–14) anos em todos os pacientes. A ADM do quadril no acompanhamento final melhorou significativamente em relação à flexão média, rotação interna (RI) e externa (RE) em 90° de flexão. Após a cirurgia, o ângulo médio de flexão foi de 91 ± 17,24° (65–120°), o ângulo médio de RI foi de 28 ± 6,5° (15–30°) e o ângulo médio de RE foi de 27,3 ± 5,3° (20–35°).

De acordo com os resultados do HHS, 4 pacientes obtiveram resultados clínicos excelentes (≥ 90 pontos), 7 bons (80–90) e um cada obteve um resultado razoável (70–80) ou ruim (< 70). No pré-operatório, o HHS médio foi de 69,2 ± 5,9 (61–78); no pós-operatório, obteve melhora para 86 ± 7,3 (75–95), com *p* < 0,00001.

O MdA pós-operatório foi classificado como bom (15–17) em 8 pacientes, regular (13 ou 14) em 6, e ruim (<13) em 2 pacientes. No acompanhamento mais recente, o MdA médio melhorou de 12,47 ± 1,13 (11–14) pontos no pré-operatório para 14,27 ± 1,83 (10–17) no pós-operatório, com *p* < 0,00001.

A anatomia radiográfica do fêmur proximal melhorou em todos os pacientes após a osteotomia modificada de Dunn. Entre os períodos pré e pós-operatório, o ângulo de deslizamento médio de Southwick melhorou de 56,60 ± 12,86° (39–76°) para 16,40 ± 4,69° (5–25°), com *p* < 0,00001. Da mesma forma, o ângulo alfa médio melhorou de 101,87 ± 12,88° (85–125°) para 29,33 ± 7,29° (20–40°), com *p* < 0,00001. O paciente número 10 ( Fig. 2a–f ) apresentou desfechos radiográficos e clínicos excelentes.

**Fig. 2:** ( **a** ) Radiografia pré-operatória em perna de rã de menino de 14,4 anos; projeção em perfil, demonstrando deslizamento de grau moderado no quadril esquerdo. ( **b** ) Imagem axial de tomografia computadorizada mostrando deslocamento da epífise femoral capital. ( **c–d** ) Projeção anteroposterior e em perna de rã em perfil imediatamente após a cirurgia. ( **e–f** ) Radiografias anteroposterior e em perna de rã em perfil tiradas 4,3 anos após a osteotomia modificada de Dunn.

A Tabela 2 resume as avaliações dos parâmetros clínicos e radiológicos realizadas antes da cirurgia e na última consulta de acompanhamento.

**Tabela 2:** Desfechos clínicos radiológicos

Parâmetro	Pré-operatório (média)	Pós-operatório (média)	Correção média	Valor de *p*	Valor de *t*	Complicações
Desfechos clínicos						Necrose avascular ( *n* = 1), infecção profunda ( *n* = 1)
ADM						
Flexão	61,67	91,00	29,33	< 0,00001	15,59	
RE em flexão	52,67	27,33	25,34	< 0,00001	12,42	
RI em flexão	0	28,00	28	< 0,00001	16,7	
HHS	69,20	86	16,8	< 0,00001	19,85	
Escore de MdA	12,47	14,27	1,8	< 0,00001	6,87	
Desfechos radiográficos						
Ângulo de deslizamento de Southwick	56,60	16,40	40,2	< 0,00001	15,07	
Ângulo alfa	101,87	29,33	72,54	< 0,00001	36,10	

**Abreviaturas:** ADM, amplitude de movimento; HHS, escore do quadril de Harris (Harris hip score); MdA, escore de Merle d'Aubignè; RE, rotação externa; RI, rotação interna.

As duas principais complicações pós-operatórias foram infecção profunda e NAV da cabeça femoral. Uma infecção profunda tratada com desbridamento e exploração da articulação do quadril resultou em rigidez articular. Um paciente com EEPF bilateral grave e hipotireoidismo teve um desfecho desfavorável após desenvolver NAV na articulação do quadril esquerdo e colapso parcial da cabeça femoral 12 semanas após a cirurgia ( Fig. 3a, b ). O paciente foi aconselhado a agendar exames de rotina e uma artroplastia total de quadril após maturidade esquelética. Nenhum outro paciente apresentou condrólise, ossificação heterotópica (OH), falência do implante, sintomas de IFA, ou OA precoce após a cirurgia.

**Fig. 3:** ( **a** ) Radiografia anteroposterior de um menino de 15,3 anos com escorregamento epifisário proximal do fêmur grave no lado esquerdo, tirada antes da cirurgia. ( **b** ) Após a osteotomia modificada de Dunn, uma radiografia de acompanhamento de 2,5 anos revelou sinais de necrose avascular na articulação do quadril esquerdo.

## Discussão

A história natural da EEPF mostrou que à medida que o grau de deslizamento aumenta, há uma deterioração perceptível e progressiva da articulação do quadril. 17 O IFA é provocado pela alteração da anatomia femoral proximal devido à proeminência metafisária anterolateral. Isso é associado a danos condrolabrais, redução da ADM, aumento da dor no quadril e OA precoce do quadril. 18

Os avanços nas técnicas cirúrgicas foram significativos e procedimentos reconstrutivos articulares mais agressivos surgiram recentemente para preservar a vascularização da cabeça femoral na EEPF, também oferecendo maior correção anatômica da deformidade. Restaurar a função normal do quadril e, simultaneamente, abordar a deformidade metafisária residual e da estabilidade fisária tem sido relatada como um possível resultado positivo da osteotomia modificada de Dunn.

Esse procedimento foi descrito pela primeira vez por Leunig et al. usando uma técnica de luxação cirúrgica segura que permitia o monitoramento intraoperatório contínuo do suprimento sanguíneo epifisário. Todos os 30 pacientes foram submetidos à redução anatômica bem-sucedida e nenhum desenvolveu NAV da cabeça femoral, de acordo com seus relatos positivos. 19

Ziebarth et al. 20 acompanharam 40 pacientes de duas instituições com EEPF moderado a grave por 1 a 3 anos. O procedimento de Dunn modificado levou à replicação da taxa de NAV ou condrólise em 0% e os autores relataram boa taxa de sucesso.

Utilizando o procedimento de Dunn modificado, Madan et al. 21 relataram o tratamento de 28 pacientes com EEPF grave. O HHS modificado médio no último acompanhamento (média: 38,6 meses) foi de 89,1, e a média do escore do quadril não artrítico (NAHS, nonarthritic hip score, em inglês) foi de 91,3. No último acompanhamento, o ângulo de deslizamento lateral foi corrigido em média 50,9° e a ADM era quase normal. Além disso, dois pacientes apresentavam NAV comprovadamente pré-existente e os outros dois a adquiriram após a cirurgia. Não foram observadas ausência de consolidação, falência do implante, infecção, trombose venosa profunda (TVP), ou OH entre os pacientes.

Entre os 43 pacientes com EEPF tratados por Upasani et al., 22 37 (86%) apresentaram escorregamento grave; 16 (37%) apresentaram 22 complicações; 10 (23%) apresentaram NAV da cabeça femoral, 4 pacientes (9%) desenvolveram pseudartrose do colo femoral e necessitaram de cirurgia de revisão; e 2 (5%) apresentaram luxação do quadril após a cirurgia.

Abdelazeem et al. 23 conduziram um estudo prospectivo após o procedimento de Dunn modificado em 31 pacientes (32 quadris) com EEPF moderado a grave. Após o tratamento, todas as medidas clínicas e radiológicas mostraram melhora significativa em um período médio de acompanhamento de 24,1 meses. As principais complicações após a cirurgia incluíram um caso de NAV pós-operatória com colapso completo da cabeça femoral e artrite, bem como um caso de infecção profunda.

Elmarghany et al. 24 relatou uma série de casos prospectiva com 30 pacientes (32 quadris) com EEPF estável crônico. No total, 30 quadris (94%) tiveram desfechos clínicos e radiográficos bons e excelentes durante um período médio de acompanhamento de 14,5 meses em relação aos parâmetros radiográficos e à função do quadril. Entretanto, 3 pacientes apresentaram NAV pós-operatória, 1 paciente teve uma redução deficiente que exigiu revisão e 1 paciente apresentou uma infecção profunda no pós-operatório. Os desfechos clínicos variaram de razoáveis a ruins.

Lerch et al. 25 usou o procedimento de Dunn modificado para tratamento de 46 pacientes (46 quadris) com EEPF grave. Desse total, 2 quadris (5%) apresentaram NAV da cabeça femoral e necessitaram de uma nova cirurgia; 3 outros quadris (7%) precisaram de revisão do implante; e, em um quadril (2%), houve progressão da OA. No acompanhamento de 10 anos, a taxa de sobrevida cumulativa foi de 86%.

O procedimento de Dunn modificado foi utilizado por Ebert et al. 26 para tratar 15 pacientes com EEPF grave. Dentro desse total, 10 pacientes obtiveram bons resultados em um acompanhamento médio de 3,8 anos, de acordo com a análise dos desfechos clínicos e funcionais (HHS >80). Em 10 pacientes, a qualidade de vida segundo o perfil de saúde de Nottingham (NHP, do inglês Nottingham health profile) foi considerada boa. Entretanto, 4 (26%) dos 15 pacientes desenvolveram NAV.

Zuo et al. 27 avaliaram retrospectivamente os resultados de 20 pacientes (21 quadris) com EEPF grave. Desse total, 19 pacientes apresentaram excelentes desfechos clínicos e radiográficos em termos de função do quadril e parâmetros radiográficos em um acompanhamento médio de 31,2 meses. Entretanto, 1 paciente (5%) com falência do implante teve desfecho ruim. Não houve casos de NAV, IFA, OA, fechamento da placa de crescimento, OH, ausência de consolidação trocantérica, ou disparidade no comprimento dos membros.

Utilizando uma técnica de Dunn modificada, Agashe et al. 28 trataram, de forma consecutiva, 30 quadris com EEPF moderado e grave. O resultado funcional médio do HHS aos 25,36 meses indicou que todos os quadris operados tinham ADMs quase normais; com os resultados sendo excelentes em 6 pacientes, bom em 17, regular em 6 e ruim em 1 paciente. Além disso, um quadril apresentou subluxação pós-operatória, que foi corrigida em uma cirurgia de revisão, enquanto 2 quadris (6,6%) desenvolveram NAV.

A Tabela 3 lista as referências dos estudos juntamente com o número de casos, classificações, duração do acompanhamento, desfechos clínicos e radiológicos pós-operatórios, incidência de NAV e outras complicações.

**Tabela 3:** Comparação dos dados e desfechos dos estudos incluídos

Autor	Número de quadris	Tipo de EEPF (moderada/ grave)	Acompanhamento (anos)	Desfechos clínicos pós-operatórios	Desfechos radiológicos pós-operatórios	NAV da cabeça do fêmur	Complicações
Ziebarth et al., 20 2009	40	16/19 (5 quadris sem informações)	2,6	HHS: 99,6; WOMAC: 1,2 (dor), 3 (função); MdA: 17,8	Ângulo de deslizamento: 8,6° Ângulo alfa: 40,6°	0%	HO: 2,5% Impacto residual: 2,5% Consolidação tardia: 7,5% Falência do implante: 7,5%
Madan et al., 21 2013	28	0/28	3,2	HHS modificado: 89,1; escore do quadril não artrítico: 91,3	Ângulo de deslizamento: 8°	7,1%	Condrólise: 3,6%
Upasani et al., 22 2014	43	6/37	2,6	Classificação de Dindo-Clavien: grau I, 4,6%; II, 6,9%; III, 34,5%; IV, 4,6%.	−	23%	Não consolidação do colo do fêmur: 9% Luxação do quadril: 5%
Abdelazeem et al., 23 2016	32	10/22	2	HHS: 96,3; WOMAC: 97; MdA: 16,7	Ângulo de deslizamento: 12,2° Ângulo alfa: 46,9°	3,1%	Infecção profunda: 3,1%
Elmarghany et al., 24 2017	32	11/21	1,2	HHS: 96,2; WOMAC: 3,3; MdA: 16,8; desfecho de Heyman e Herdon: excelente ou bom, 93,7%	Ângulo de deslizamento: 5,6° Ângulo alfa: 51,1°	9,3%	Infecção profunda: 3% Revisão: 3%
Lerch et al., 25 2019	46	0/46	9	HHS: 94; MdA: 17; HOOS: 91	Ângulo de deslizamento: 7°	5%	OA: 2%, Falência do implante: 7%
Ebert et al., 26 2019	15	0/15	3,8	HHS: 85,7 NHP: 0,91 VAS: 1,6	−	26,6%	Subluxação do quadril: 13,3% Falência do implante: 13,3%
Zuo et al., 27 2020	21	0/21	2,7	HHS: 96,7 WOMAC: 95,4	Ângulo de deslizamento: 7,5° Ângulo alfa: 42°	0%	Falência do implante: 5%
Agashe et al., 28 2020	30	20/10	2,1	HHS: 81,8	Ângulo de deslizamento: 9,9° Ângulo alfa: 33,6°	6,6%	Subluxação do quadril: 3,3%
Estudo atual	16	6/10	8,6	HHS: 86; MdA: 14,2	Ângulo de deslizamento: 16,4° Ângulo alfa: 29,3°	6,2%	Infecção profunda: 6,2%

**Abreviaturas:** EEPF, escorregamento epifisário proximal do fêmur; NAV, necrose avascular; HHS, escore do quadril de Harris (Harris hip score); HO, ossificação heterotópica; HOOS, escore de deficiência do quadril e desfecho de osteoartrite (Hip disability and osteoarthritis outcome score); MdA, escore de Merle d'Aubignè; NAHS, escore do quadril não artrítico (nonarthritic hip score); NHP, perfil de saúde de Nottingham (Nottingham health profile); EVA, escala visual análogica; WOMAC, índice de artrite das universidades de Western Ontario e McMaster (Western Ontario and McMaster Universities arthritis index).

Neste estudo, o número de pacientes com EEPF moderado a grave tratados com a osteotomia modificada de Dunn foi semelhante ao relatado na literatura. No último acompanhamento, foram registradas duas complicações (NAV e infecção profunda), mas também melhoras notáveis na ADM, HHS médio e MdA médio. Esses achados indicam que a osteotomia modificada de Dunn pode ser usada para realinhamento da EEPF com fise aberta e um baixo risco de complicações.

No tratamento das formas moderadas a graves de EEPF, a osteotomia modificada de Dunn é uma técnica cirúrgica segura e confiável que deve ser fortemente considerada. Com uma taxa de complicações controlável, este procedimento pode restaurar a anatomia femoral proximal, abordando o ângulo de deslizamento e o deslocamento cabeça-colo, ajudando a manter a função normal do quadril. 29

Os pontos fortes deste estudo incluem o uso de uma população bem-definida de pacientes com EEPF moderado a grave e desfechos funcionais e radiológicos pós-operatórios que estão positivamente correlacionados a resultados bem-sucedidos em longo prazo. Este estudo tem várias limitações, como o pequeno tamanho da amostra, o desenho retrospectivo do estudo de coorte única, o potencial viés de seleção devido à ausência de randomização e o delineamento experimental não comparativo, sem grupo controle.

## Conclusão

A osteotomia modificada de Dunn, ao preservar meticulosamente o retalho retinacular posterior de tecido mole, auxilia o realinhamento anatômico de uma epífise femoral capital deslocada, mantendo a perfusão da cabeça femoral. A execução bem-sucedida deste procedimento reconstrutivo complexo com desfechos favoráveis em longo prazo requer a compreensão precisa da anatomia vascular do quadril e o conhecimento completo da técnica cirúrgica.
